# Supplementary material for: Reducing Barriers for Best Practice in People Living with Dementia: Cross-Cultural Adaptation and Content Validity of the Brazilian Version of the Pain Assessment in Impaired Cognition (PAIC-15) Meta-Tool
Source: Int J Environ Res Public Health. 2025 Aug 26;22(9):1324. doi: 10.3390/ijerph22091324 (PMC12469513; doi:10.3390/ijerph22091324)
Supplement: Supplementary file 1 [file ijerph-22-01324-s001.zip › ijerph-3700839-Supplementary File S1.pdf]

Nome do paciente: Data:

Pain Assessment in Impaired Cognition (PAIC -15)

Item	Significado do Item	Não presente	Grau leve	Grau moderado	Grau intenso	Não aplicável/ não quantificável
**EXPRESSÃO FACIAL**						
**Franzindo a testa**	Abaixando e/ou contraindo as sobrancelhas	**0**	**1**	**2**	**3**	x
**Apertando os olhos**	Apertando os olhos com tensão ao redor dos mesmos	**0**	**1**	**2**	**3**	x
**Levantando o lábio superior**	Levantando o lábio superior e/ou franzindo o nariz	**0**	**1**	**2**	**3**	x
**Abrindo a boca**	A boca está aberta, a mandíbula caída	**0**	**1**	**2**	**3**	x
**Parecendo tenso**	A face mostra tensão ou preocupação	**0**	**1**	**2**	**3**	x
**MOVIMENTOS CORPORAIS**						
**Enrijecido ou rígido**	Petrificado, evitando movimento, segurando a respiração	**0**	**1**	**2**	**3**	x
**Postura de proteção**	Protegendo a área afetada, segurando parte do corpo, evitando o contato, movimento de retirada	**0**	**1**	**2**	**3**	x
**Resistindo ao cuidado**	Resistindo em se mover ou resistindo ao cuidado, não cooperativo	**0**	**1**	**2**	**3**	x
**Fricção**	Esfregando ou massageando a área afetada	**0**	**1**	**2**	**3**	x
**Inquietação**	Inquieto, torcendo ou esfregando as mãos, balançando para frente e a para trás	**0**	**1**	**2**	**3**	x
**VOCALIZAÇÃO**						
**Usando as palavras relacionadas à dor**	Usando palavras como, “ai”, “ui”, “isso dói”	**0**	**1**	**2**	**3**	x
**Gritando**	Expressando palavras em voz alta	**0**	**1**	**2**	**3**	x
**Gemendo**	Produzindo som abafado e sem articulação	**0**	**1**	**2**	**3**	x
**Resmungando**	Pronunciando palavras ou sons indistintos	**0**	**1**	**2**	**3**	x
**Reclamando**	Expressando estar infeliz, doente, desconfortável e/ou com dor	**0**	**1**	**2**	**3**	x
	**SOMA =**					

Em qual situação você observou o indivíduo?

☐ Em repouso

☐ Durante uma atividade de vida diária (AVD), por favor descreva:

☐ Durante um momento passivo, por favor descreva:

PAIC15
